# A hotspot of diversity: novel Shewanella species isolated from Baltic Sea sediments delineate a sympatric species complex

**DOI:** 10.1099/ijsem.0.006480

**Published:** 2024-08-16

**Authors:** Alberto J. Martín-Rodríguez, Víctor Fernández-Juárez, Valerie D. Valeriano, Indiwari Mihindukulasooriya, Livia Ceresnova, Enrique Joffré, Susanne Jensie-Markopoulos, Edward R. B. Moore, Åsa Sjöling

**Affiliations:** 1 Department of Clinical Sciences, University of Las Palmas de Gran Canaria, Las Palmas de Gran Canaria, Spain; 2 Centre for Translational Microbiome Research (CTMR), Department of Microbiology, Tumor and Cell Biology, Karolinska Institute, Stockholm, Sweden; 3 Department of Infectious Diseases, Institute for Biomedicine, Sahlgrenska Academy of the University of Gothenburg, Gothenburg, Sweden; 4 Culture Collection University of Gothenburg (CCUG), Sahlgrenska University Hospital and Sahlgrenska Academy of the University of Gothenburg, Gothenburg, Sweden; 5 Department of Chemistry and Molecular Biology (CMB), University of Gothenburg, Gothenburg, Sweden; 6 Department of Clinical Microbiology, Sahlgrenska University Hospital, Region Västra Götaland, Gothenburg, Sweden

**Keywords:** Baltic Sea, phylogenomics, *Shewanella*, sediment bacteria, species complex

## Abstract

Two bacterial strains, SP1S1-4^T^ and SP2S1-2^T^, were isolated from sediment samples collected in the Stockholm archipelago in November 2021. Following whole-genome sequencing, these strains were identified as tentatively belonging to two novel *Shewanella* genospecies, based on digital DNA–DNA hybridization, as implemented in the Type Strain Genome Server. *Shewanella septentrionalis*, *Shewanella baltica* and *Shewanella hafniensis* were, in this order and within a narrow genomic relatedness range, their closest genotypic relatives. Additional sampling and sequencing efforts led to the retrieval of distinct isolates that were monophyletic with SP1S1-4^T^ and SP2S1-2^T^, respectively, based on phylogenomic analysis of whole-genome sequences. Comparative analyses of genome sequence data, which included blast-based average nucleotide identity, core genome-based and core proteome-based phylogenomics, in addition to MALDI-TOF MS-based protein profiling, confirmed the distinctness of the putative novel genospecies with respect to their closest genotypic relatives. A comprehensive phenotypic characterisation of SP1S1-4^T^ and SP2S1-2^T^ revealed only minor differences with respect to the type strains of *S. septentrionalis*, *S. baltica* and *S. hafniensis*. Based on the collective phylogenomic, proteomic, and phenotypic evidence presented here, we describe two novel genospecies within the genus *Shewanella*, for which the names *Shewanella scandinavica* sp. nov. and *Shewanella vaxholmensis* sp. nov. are proposed. The type strains are, respectively, SP2S1-2^T^ (=CCUG 76457^T^=CECT 30688^T^), with a draft genome sequence of 5 041 805 bp and a G+C content of 46.3 mol%, and SP1S1-4^T^ (=CCUG 76453^T^=CECT 30684^T^), with a draft genome sequence of 4 920147 bp and a G+C content of 46.0 mol%. Our findings suggest the existence of a species complex formed by the species *S. baltica*, *S. septentrionalis*, *S. scandinavica* sp. nov., and *S. vaxholmensis* sp. nov., with *S. hafniensis* falling in the periphery, where distinct genomic species clusters could be identified. However, this does not exclude the possibility of a continuum of genomic diversity within this sedimental ecosystem, as discussed herein with additional sequenced isolates.

## Introduction

*Shewanella* species have a highly versatile metabolism, which facilitates the occupation of diverse environmental and host-associated niches and contributes to their ubiquitous distribution, primarily within aquatic ecosystems [1,2]. The adaptability of *Shewanella* species is supported by a broad repertoire of metabolic capacities including the ability to use a wide array of electron acceptors for respiration [2,4]. Because of these features, redox-stratified environments, such as humid sediments, have intrinsic characteristics to represent ‘hotspots’ for the occurrence of *Shewanella* species. Indeed, our recent study that resolved the taxonomy of *Shewanellaceae*, based on whole-genome sequence (WGS) phylogenomic analysis, revealed the existence of numerous novel genospecies among sequenced isolates, most of which had been isolated from this environmental source [5].

The large genetic variation within bacterial populations represents a challenge for the definition of bacterial species. DNA–DNA hybridization (DDH) has traditionally represented the ‘gold standard’ for species circumscription, with a value of ≥70 % defining the cutoff for species designation [6]. In the current genomic era, so-called because of the widespread implementation of whole genome sequencing in systematics, bacterial species classification relies largely on genomic relatedness. Empirical DDH determinations have been replaced by *in silico*-calculated parameters, namely digital DNA–DNA hybridization (dDDH) and diverse implementations of average nucleotide identity (ANI) algorithms, demonstrating that the traditional 70 % DDH threshold correlates with 70 % dDDH and 95–96 % ANI, respectively [7,9]. Despite the availability (or perhaps because) of these valuable bioinformatics tools, a heated debate persists over whether genetic discontinuity boundaries exist to delineate bacterial species, in spite of the availability of thousands of bacterial genomes representing species native to a broad range of environments [10,12].

In this study, we collected sediment samples from a region of the Baltic Sea in search of *Shewanella* species. We report the isolation of two *Shewanella* strains, SP1S1-4^T^ and SP2S1-2^T^, which were analysed for their phylogenetic and taxonomic relationships with other species of *Shewanella* by in depth genome sequence analyses, MALDI-TOF MS-based protein profiling, and phenotyping. Through additional sampling and genome sequence analysis efforts, we interrogated whether these and other coexisting, phylogenetically related strains formed distinct genomic clusters or rather represented a continuum of diversity with blurry, unclear taxonomic borders. Altogether, these strains present a sample of genomic diversity of a bacterial taxon within a particular environment and a ‘snapshot’ of the evolution of *Shewanella* speciation.

## Methods

### Bacterial sampling and isolation

Sediments were collected on the island of Vaxön on two sampling occasions. On the first sampling campaign (14 November 2021), two sampling points were established (59° 24′ 19.9″ N 18° 21′ 08.5″ E; 59° 24′ 03.6″ N 18° 20′ 18.7″ E). Superficial shore sediments were scooped out, using sterile 50 ml tubes, and stored at ambient temperature for 3–4 h upon transportation to the laboratory. Samples were processed as recently described [13]. In brief, upon arrival, the tubes containing sediment samples were centrifuged (54 *g*, 4 °C) and the supernatant water collected during sampling was decanted. Sediment samples of approximately 15 g were separated and overlaid with 30 ml 0.22 µm filtered water from the collection site, tilt-shaken for 1 h (100 r.p.m., 60° angle, 4 °C), serially diluted in sterile PBS (0.01 M, pH 7.4), and plated on Lyngby’s iron agar (Oxoid) supplemented with 0.04 % w/v l-cysteine. Agar plates were incubated at 26–28 °C and black colonies, indicative of H_2_S production, were selected as *Shewanella*-like bacteria and re-streaked three times on the same medium for purification. Identification to the genus level was achieved by matrix-assisted laser desorption/ionization-time of flight (MALDI-TOF) mass spectrometry (MS) analysis (MALDI Biotyper Sirius System, Bruker). *Shewanella* strains were found to grow satisfactorily in Miller’s lysogeny broth (LB; supplied by the substrate unit at the Karolinska University Hospital) and marine broth 2216 (Difco), and were grown in the former for cryopreservation at −80 °C upon addition of glycerol to a final concentration of 20 % (v/v). On the second sampling occasion (12 August 2023), sediment cores were collected (59° 24′ 01.0″ N 18° 20′ 20.2″ E) using cutoff sterile 30 ml syringes (VWR 613–2035) as described elsewhere [14]. Sediment material from different depths was taken for bacterial isolation as described above, except that the initial centrifugation step to remove overlaying water was not needed, and filtered water from the collection site was replaced by sterile PBS.

### Genomic DNA extraction and sequencing

Genomic DNA was extracted from overnight cultures using the DNeasy Blood and Tissue kit (Qiagen) and sequenced, as previously described [5]. In brief, 50 ng gDNA was employed for library preparation, using the MGIEasy FS Library Prep Set (MGI Tech), following the manufacturer’s recommendations. Equimolar pooled libraries were used for DNA circularization, using the MGIEasy Circularization Kit (MGI Tech), and sequenced (2×100 bp paired-ends) on an NBSEQ-G400 instrument (MGI Tech). Genomes were assembled using SPAdes version 3.15.2, as implemented in Bactopia version 1.7.x [15] (strains SP2S1-2^T^, SP1S1-4^T^, SP1S1-7, SP1S2-4, SP2S2-4, and SP2S2-6, collected on the first sampling occasion), or SPAdes version 3.15.5, as implemented in BACTpipe (https://github.com/ctmrbio/BACTpipe; strains VAX-SP4-0CM-7, VAX-SP4-0CM-4, VAX-SP0-4CM-4, and VAX-SP0-0CM-1, collected on the second sampling occasion).

Near-complete sequences of 16S rRNA genes were obtained by PCR amplification followed by Sanger DNA sequencing, using previously reported amplification and sequencing primers [16].

### Bioinformatic analyses of WGS data and 16S rRNA gene sequences

Phylogenomic reconstructions based on dDDH were carried out using the Type Strain Genome Server (TYGS) [17,18], available at https://tygs.dsmz.de. TYGS phylogenomic reconstructions were inferred with FastME 2.1.6.1 [19] from Genome blast Distance Phylogeny (GBDP) distances calculated from genome assemblies and the trees were rooted at the midpoint [20]. blast-based ANI (ANIb) calculations were performed with JSpeciesWS [21], available at https://jspecies.ribohost.com/jspeciesws. Core genome-based analyses were conducted with Panaroo version 1.3.4 [22]. Prior to the analysis, Prokka version 1.14.6 [23] was employed to annotate the genomes. Panaroo was run in strict mode, and gene clustering was made using the default parameters, i.e., core threshold (0.98), sequence identity threshold (0.98), and length difference cutoff (0.98). This means that sequences must have a similarity or identity score of at least 98 % to be considered as the same gene cluster and must have a length difference of no more than 2 % to be considered similar, respectively. Paralogous genes were merged. Core genome-based phylogenomic trees were then reconstructed with FastTree version 2.1.10 [24] using 1000 bootstraps replications, midpoint rooted, and visually represented with iTOL version 6 [25] (https://itol.embl.de).

Pairwise average amino-acid identity (AAI) values, based on whole proteomes, were calculated using the EzAAI pipeline [26]. For core proteome-based analysis, a pangenome was obtained with anvi’o version 8 [27], following the pangenomics workflow (https://merenlab.org/2016/11/08/pangenomics-v2). Briefly, the study involved running two scripts: anvi-gen-contigs-database to create a database, utilizing Prodigal version 2.6.3 for identifying open reading frames within contigs [28]; and anvi-run-ncbi-cogs to annotate genes using NCBI’s Clusters of Orthologous Groups visualization [29,30]. Anvi'o employs diamond to compute the similarity between each amino acid sequence within every genome against all other sequences across the genomes [31]. Following this, the Markov cluster algorithm was employed to identify clusters within the obtained amino acid sequence similarity results [32]. Anvi-get-sequences-for-hmm-hits was used to retrieve the sequences of single-copy gene clusters present in all genomes. ANIb among genomes was computed using PyANI via anvi-compute-ani [33], and the presence or absence of gene clusters within each genome was graphically depicted. The concatenated amino acid sequences corresponding to these gene clusters were extracted and a phylogenomic tree was reconstructed with FastTree v2.1.10 using 1000 bootstraps replications [24], midpoint rooted, and visually represented with iTOL v6 [25].

Maximum-likelihood (ML), neighbour-joining (NJ) and maximum-parsimony (MP) phylogenies, as well as pairwise sequence similarities of partial 16S rRNA gene sequences, were computed using mega X [34]. For the ML reconstruction, the best-fit model of nucleotide substitution (HKY+G+I), as determined by the software, was employed. All phylogenies included 1000 bootstrap replicates.

### MALDI-TOF MS protein profiling and main spectra profile (MSP) analysis

#### Protein extraction

Protein extraction from environmental *Shewanella* species and three reference type strains, *Shewanella baltica* CECT 323^T^, *Shewanella hafniensis* ATCC BAA-1207^T^, and *Shewanella septentrionalis* CCUG 76164^T^, was performed according to the Bruker MALDI Biotyper Protocol. In brief, a suspension of fresh colony biomass in 300 µl HPLC-grade water was denatured by adding 900 µl absolute ethanol. The resulting mix was centrifuged to remove the supernatant, further centrifuged to eliminate excess ethanol, and the remaining pellet dried at room temperature. Optimal lysis was achieved by adding 25 µl 70 % formic acid (v/v) and 25 µl 100 % acetonitrile, followed by mixing and centrifugation. The obtained supernatant (1 µl) was spotted onto an MBT Biotarget 96 plate (#1 840 375, Bruker) in eight replicate wells. Dried spots were immediately overlaid with 1 µl HCCA matrix solution (α-cyano-4-hydroxycinnamic acid, #8255344, Bruker Daltonics).

#### Raw spectrum acquisition and processing

A bacterial test standard (BTS) was used for calibration and quality control during spectral library acquisition. The same BTS control was used for raw peak pre-processing, including subtraction, smoothing, and peak recalibration, ensuring consistent mass calibration constants. Protein mass spectra from 2 to 21 kDa were acquired in a linear positive mode with a laser frequency of 200 Hz. Instrument settings included ion source 1 at 20 kV, ion source 2 at 18.22 kV, lens at 6.01 kV, and linear detector at 2811 V. Spectra from each spot were automatically acquired using the flexControl programme (Bruker Daltonics), with 240 laser shots in total, 40 shots at six automatically selected positions per spot. Thirty-two spectral peaks were acquired per *Shewanella* strain and selected to fulfil more than 24 spectral peaks per strain for the initial main spectra profile (MSP) library creation.

#### Data analysis

A composite correlation index (CCI) database was created using the Biotyper Standard Method to assess and curate spectral reproducibility. A subset of the MSP libraries with at least 20 spectral peaks per strain was selected, containing highly similar spectral data based on CCI score matches. MSP library subsets were then further analysed using multivariate cluster analysis. Using ClinProTools 3.0 (https://bruker-daltonics-clinprotools.software.informer.com/3.0/), a principal component analysis (PCA) was generated with the unit variance scaling method, where the intensity of each peak will be divided by the standard deviation for this peak in the data set. Further, a spectral view resolution and heatmap of the MALDI-TOF normalized peak intensities was generated. Significant peaks were sorted by the ClinProTools software based on the difference between the maximal and minimal peak area/intensity for all classes (termed DAve), as well as on the p-values of several statistical tests of average peak intensities including the t-test (PTTA), Wilcoxon/Kruskal–Wallis test (PWKW), and Anderson-Darling test (PAD). Differences were considered significant when PAD and PWKW were both <0.05, or when PAD ≥ 0.05 and PTTA <0.05.

Finally, a dendrogram based on the final MSP databases was generated by the MBT Compass Explorer (Bruker Daltonics) using the Biotyper MSP dendrogram creation standard method with score-oriented, average linkage method, with Spearman’s correlation distance measure to mitigate the influence of potential outliers [35]. Distinct peaks between strains were identified based on significant p-values from the Wilcoxon/Kruskal–Wallis Test (PWKW<0.000001). Validation was performed for the final MSP database through iterative comparison between the database and the collected spectra for each strain.

### Analysis of phenotypic properties and enzymatic reactions

The capacity of bacterial strains to grow on different media was tested on blood agar medium, Miller’s LB agar or broth, and marine agar or broth (Difco 2216). Bacterial growth at different temperatures (4, 23, 28, 30, 32, 35, 37, and 42 °C) was tested on Miller’s LB agar. Growth at different NaCl concentrations was tested on LB agar supplemented with NaCl to a final concentration of 1.0, 2.5, 5.0, 7.5, or 10.0 % (w/v), or without NaCl supplementation, at 28 °C. Growth under anaerobic conditions was tested at 28 °C on Miller’s LB agar plates in jars, with anaerobiosis induced by AnaeroGen sachets (Oxoid, Thermo Fisher Scientific). Growth at different pH values (pH 4.5–10.5, at 1.0 pH unit intervals) was tested in buffered LB broth at 28 °C, as previously described [13], by addition of 50 mM of the following buffers: acetic acid–acetate (pH 4.5 and 5.5); MES (pH 6.5); HEPES (pH 7.5); Tris–HCl (pH 8.5); and carbonate–bicarbonate (pH 9.5 and 10.5). Growth in 96-well plates containing buffered media was automatically recorded every 15 min as OD_600_, using a SpectraMax i3x microplate reader, with shaking between measurements, and growth curves were visualized with GraphPad Prism version 10. The analysis of metabolic features was carried out using the CCUG NFX worksheet (https://ccug.se/documents/worksheets/nfx.pdf).

### Microscopy

Gram staining was performed using a commercial kit (Millipore, Ref. 77730) on cells grown on Miller’s LB agar. Visualization of individual cells by scanning electron microscopy (SEM) was conducted using a Zeiss Ultra 55 instrument upon fixation of cells grown in Miller’s LB broth by resuspension in a solution of 2.5 % glutaraldehyde (v/v) and 1 % paraformaldehyde (v/v) in 0.1 M phosphate buffer, pH 7.4, and critical point drying (Leica EM CPD 030). The determination of cell dimensions (average length and width) was performed with FiJi Bio-Formats [36], taking 15 arbitrarily chosen individual cells of each strain.

## Results and discussion

### Isolation and ecology of Baltic Sea sediment native *Shewanella* species

Sediments were collected from sampling points located on Vaxön, a small island located at the boundary between the outer and inner Stockholm archipelago, which is an enclosed system characterized by low water salinity, a high input of freshwater from Lake Mälaren, and regular ice formation during winter [37,38]. The town of Vaxholm, located on the island, is a popular summer destination in Sweden and an entry point to the outer Baltic Sea, which implies a certain level of anthropic influence. The two sampling occasions conducted as part of this study, carried out in late fall and summer, respectively, and separated approximately 2 years in time, led to the straightforward retrieval of numerous *Shewanella* solates on Lyngby’s iron agar supplemented with l-Cys, as confirmed by MALDI-TOF MS identification, indicating that *Shewanella* species are abundant, non-transient members of the indigenous sedimental microbiota in this region. The study presented here began with the obtaining of the WGS of representative isolates and their taxonomical classification.

### Genome relatedness indexes and WGS-based taxonomy

Strains SP1S1-4^T^ and SP2S1-2^T^, retrieved from the first sampling occasion in November 2021, were found to represent putative novel species, as inferred by dDDH, upon submission of the assembled genome sequences to TYGS, which contains a complete genome sequence database with all known species in the family *Shewanellaceae* after recent efforts involving the genome sequencing of all type strains lacking a WGS in public repositories, as this had not been a requisite for the description of novel taxa until relatively recently [5]. The basic genomic features of strains SP1S1-4^T^ and SP2S1-2^T^ are summarized in Table 1. Table S1 summarizes the pairwise dDDH comparisons between the set of five strains composed by the two novel isolates and the type strains of the most closely related species, inferred by the genome blast distance phylogeny (GBDP) distance formula *d*_4_ implemented in TYGS, which is the recommended formula for draft genome sequences, as it does not consider genome length [8]. Even though TYGS highlighted both strains as representatives of two potentially novel species based on this overall genome-relatedness index (OGRI), it was obvious that the two strains were closely related between each other (dDDH 64.9 %), as well as with the type strains of *Shewanella septentrionalis* (dDDH 64.8 and 63.8 % for SP1S1-4^T^ and SP2S1-2^T^, respectively) and *Shewanella baltica* (dDDH 61.3 and 63.2%, for SP1S1-4^T^ and SP2S1-2^T^, respectively). The type strain of *Shewanella hafniensis* was more distant from both strains, with dDDH 60.3 and 59.1 % for SP1S1-4^T^ and SP2S1-2^T^, respectively. In view of these data, we employed a second OGRI, ANIb, as implemented in JSpeciesWS [21]. ANIb is considered the reference implementation of the ANI algorithm and was preferred over the MUMmer-based ANI algorithm (ANIm), as the latter is considered less adequate for draft genome sequences [39]. The threshold for species designation is typically set at ANI values of 95–96 % [10], although this threshold window is somewhat variable across genera and depends on the implementation of the ANI algorithm being used. For instance, values as low as ANIb of 93 % have been employed to support certain species delineations within the genus *Streptococcus* [40], whereas values as high as ANIm 96.7 % supported species circumscriptions within the genus *Streptomyces* [41]. Although dDDH has been claimed to outperform distinct implementations of ANI [18], the latter constitutes an alternative OGRI that is, together with dDDH, recommended for bacterial systematics [42,43]. The pairwise ANIb distance matrix obtained for SP1S1-4^T^ and SP2S1-2^T^ and the type strains of *S. hafniensis*, *S. baltica* and *S. septentrionalis* is presented in Table S2. The calculated pairwise ANIb values could discriminate strains SP1S1-4^T^ and SP2S1-2^T^ from the type strain of *S. hafniensis*, the most distantly related of the three reference type strains (ANIb <95 %). With respect to the type strain of *S. baltica*, only strain SP1S1-4^T^ presented a pairwise ANIb value <95 %. Pairwise comparisons in all other cases, that is, each of the novel isolates with respect to the type strain of *S. septentrionalis* or with respect to each other, fell within the 95–96 % window, which is known to be a ‘transition zone’ in which taxonomic boundaries at the specific level may be unclear [9].

**Table 1.** Basic characteristics of the genome sequences of strains SP2S1-2^T^ and SP1S1-4^T^

Parameter	SP2S1-2^T^	SP1S1-4^T^
Genome accession (GenBank)	JAUOES000000000	JAUOEV000000000
Genome size (bp)	5 041 805	4 920 147
G+C content (mol%)	46.3	46.0
Genome coverage	82×	90×
No. contigs	71	53
Contig N50 (kb)	189.8	164.1
Contig L50	10	10
Total genes	4469	4261
Coding sequences	4328	4121
RNA genes	109	96
Pseudo-genes	32	44
Completeness (%)*	99.5	99.5
Contamination (%)*	0.15	0
dDDH (%)†	63.8	64.8
ANIb (%)†	95.4	95.6
AAI (%)†	97.5	97.5

*Assessed with CheckM version 1.2.2 [61] as implemented in NCBI GenBank.

**†Reported values are with respect to the closest genotypic relative, *Shewanella septentrionalis* SP1W3T.

A recent update of the proposed minimal standards for the use of genome data for the taxonomy of prokaryotes [43] emphasizes the need for phylogenomic congruence beyond the mere application of predetermined OGRI thresholds, which, as pointed out above, could vary depending on the taxon being studied. To shed light on the taxonomic status of strains SP1S1-4^T^ and SP2S1-2^T^, we sequenced the genomes of representative *Shewanella* isolates retrieved from a second sampling occasion in Vaxholm during the summer of 2023. Upon submission of the assembled genome sequences to TYGS, strains VAX-SP0-0CM-1 and VAX-SP0-4CM-4 were found to be putatively conspecific with strain SP2S1-2^T^, whereas strains VAX-SP4-0CM-4 and VAX-SP4-0CM-7 were found to be putatively conspecific with SP1S1-4^T^. A phylogenomic reconstruction, inferred from GBDP distances calculated from genome assemblies, is presented in Fig. S1 (available in the online version of this article) as a midpoint-rooted tree. For this reconstruction we introduced three representative genome sequences of the species *S. baltica* and *S. hafniensis*, including their respective type strains (see [5] for the reclassification of strain Pdp11 as *S. hafniensis* Pdp11), and the only genome sequence available for *S. septentrionalis*. The resulting phylogeny showed that strains SP2S1-2^T^, VAX-SP0-0CM-1, and VAX-SP0-4CM-4, on the one hand, and SP1S1-4^T^, VAX-SP4-0CM-4, and VAX-SP4-0CM-7, on the other hand, form distinct, well-supported clades that are clearly separated from the *S. baltica* and *S. hafniensis* clades and from *S. septentrionalis*. This phylogenomic reconstruction, therefore, supports the notion that SP2S1-2^T^ and SP1S1-4^T^ represent, each, a novel species within the genus *Shewanella*.

### Core genome-based and core-proteome based phylogenetic analyses

**Fig. 1.**
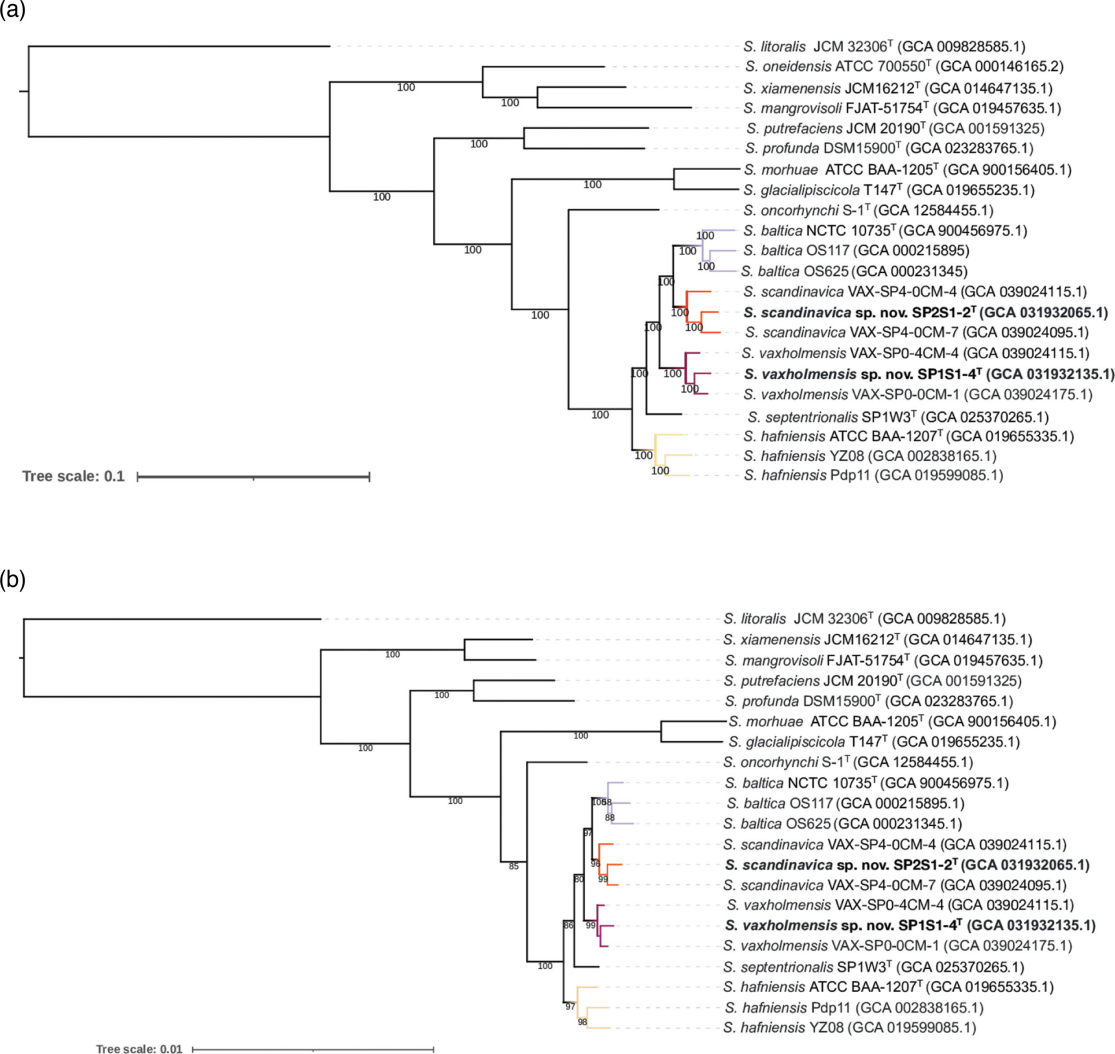
(**a**) Core genome-based and (**b**) core proteome-based phylogenies of *Shewanella* strains. Clustering was performed using 1358 single-copy core genes or 662 single-copy core proteins, respectively. Reconstructions are presented as maximum-likelihood phylogenetic trees from alignments of gene or protein sequences, respectively. GenBank accessions are indicated in parentheses. Support values≥50 are indicated on the nodes. Colours highlight well-supported subclades formed by *S. baltica*, *S. septentrionalis*, *S. hafniensis*, and the two novel species presented in this study.

**Fig. 2.**
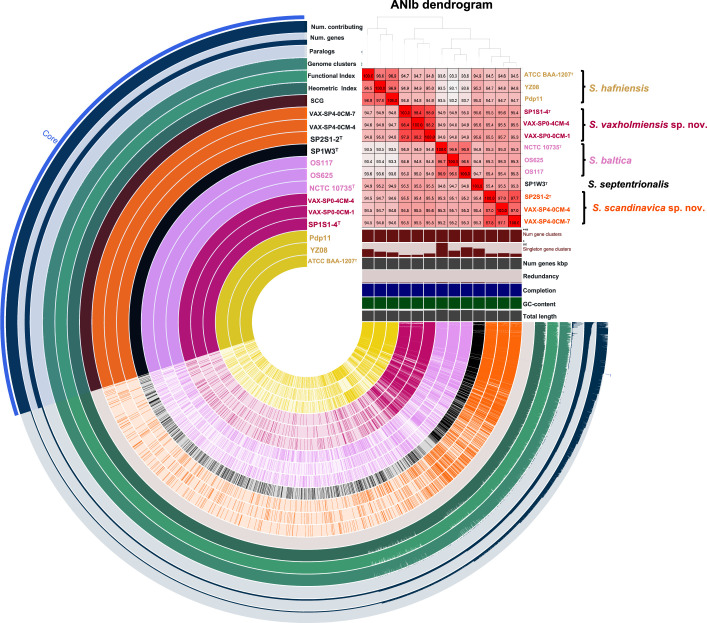
Pangenome analysis generated with anvi’o for 13 *Shewanella* genomes. The layers represent individual genomes organized by their phylogenomic relationship, i.e., based on ANIb values. A colour matrix was generated using a heatmap based on ANIb values, effectively illustrating the relationships between clusters. Same circles and label colours represent genomes belonging to the same species. In the layers, solid colours indicate the presence of a gene cluster whereas transparencies represent its absence. The pangenome was constituted by 7760 gene clusters (GCs), and the core genome was composed of 3029 gene clusters. The seven outermost layers correspond to various statistics related to the analysis, i.e., the number of contributing genomes per GC, number of genes per GC, maximum number of paralogues per GC, species core genome clusters, functional homogeneity index, heometric homogeneity index, and single core genes clusters.

If strains SP2S1-2^T^ and SP1S1-4^T^ represented two novel *Shewanella* species, it was expected that core genome and core proteome-based phylogenomic reconstructions would reflect the topology of the WGS-based phylogeny and support the same taxonomic conclusions. Hence, a core genome analysis was performed using the genome sequences of the strains shown in Fig. S1. Out of a pan genome comprising 13 220 genes, 1358 single-copy gene clusters were found to be present in all genomes, as inferred with Panaroo version 1.3.4 [22]. The topology of the resulting core genome-based tree (Fig. 1a) was coherent with that of the WGS-based phylogenomic tree (Fig. S1) and presented SP2S1-2^T^ and SP1S1-4^T^ and their conspecific strains as well-supported monophyletic taxa. Similarly, a pan-proteome inferred with anvi’o version 8 [27], defined 17 066 gene clusters, of which 662 single-copy gene clusters were found to be present in all genomes. The topology of the phylogenetic tree constructed using the concatenated amino acid sequences of the 662 single-copy protein coding genes (Fig. 1b) was highly congruent with that of the core genome-based and WGS-based reconstructions, supporting identical conclusions. In Fig. 2 we visually integrate pangenome and OGRI data (ANIb) by displaying the core and accessory genome content of 13 strains pertaining to the clade formed by the species *S. baltica*, *S. septentrionalis*, *S. hafniensis*, and the two putative novel species, along with dendrogram clustering based upon ANIb of aligned genomic regions. From this representation it can be concluded that i) conspecific strains form clusters and share ANIb values ≥96 %, ii) species within this clade are closely related genomically, with ANIb values in the range 93.1–95.7 % and sharing a high proportion of core genes (3029 single-copy gene clusters present in all genomes, out of a pangenome composed of 7760 gene clusters), and iii) the accessory genome content, which was inferred to roughly represent 40 % of the pangenome, might reflect substantial levels of lateral gene transfer [44].

### 16S rRNA gene sequence similarity and phylogeny

As a taxonomic requisite, partial 16S rRNA sequences of SP2S1-2^T^ and SP1S1-4^T^ were obtained by PCR amplification followed by Sanger DNA sequencing. The topologies of the ML (Fig. S2), NJ (Fig. S3) and MP (Fig. S4) trees reconstructed using the partial 16S rRNA gene sequences of both strains in relation to those of the type strains of a representation of *Shewanella* species, including their closest phylogenetic relatives, were all similar, with both novel strains clustering most closely with *S. septentrionalis*, albeit with a resolution that did not allow discrimination between the strains as distinct species. Indeed, strains SP1S1-4^T^ and SP2S1-2^T^ exhibited a 16S rRNA sequence similarity of 100 % with respect each other, and 99.1, 99.2 and 99.8 % against the type strains of *S. hafniensis*, *S. baltica* and *S. septentrionalis*, respectively, as computed by mega X software. These values are well beyond the conventional 98.7 % threshold established for species delineation [42]. This result was, however, not unexpected for intimately related isolates such as these. First, it has been shown that phylogenetic reconstructions based on 16S rRNA sequences lack the taxonomic resolution to accurately delineate boundaries between certain closely related species within the genus *Shewanella* [5,13, 45, 46]. Second, taxa within specific genera, such as *Aeromonas*, characterized by significant conservation of 16S rRNA gene sequences, frequently exhibit sequence similarities that pose challenges for taxonomic classification [47], spanning from 96.7 to 100 % inter-specifically [48]. Indeed, high 16S rRNA gene sequence similarity is expected from divergent, yet closely related lineages such as the ones represented by SP1S1-4^T^ and SP2S1-2^T^, with a sympatric lifestyle with respect to their closest genotypic relatives.

### Proteome-based analysis and MALDI-TOF MS protein profiling

To complement our analyses, we conducted in silico and wet lab analyses of proteome content of strains SP1S1-4^T^ and SP2S1-2^T^, starting with pairwise AAI comparisons. The type strain of *S. baltica* exhibited an AAI of 96.6 and 97.2 % with respect to the type strains of *S. hafniensis* and *S. septentrionalis*, respectively, while the two latter type strains exhibited a pairwise AAI of 97.3 %. This indicates that the threshold for species delineation within this particular clade using this index is higher than the conventionally accepted 95–96 % threshold [49]. All-against-all pairwise AAI values in the strain set composed of the two novel strains and the three reference type strains are presented in Table S3. Strains SP1S1-4^T^ and SP2S1-2^T^ presented the lowest AAI values with respect to the type strain of *S. hafniensis* (97.2 and 97.1 %, respectively), whereas pairwise comparisons with the type strains of *S. baltica* or *S. septentrionalis*, or against each other, were approximately 97.5 %. Overall, the high AAI similarity shared by species within this clade precludes taxonomic conclusions based upon this indicator.

**Fig. 3.**
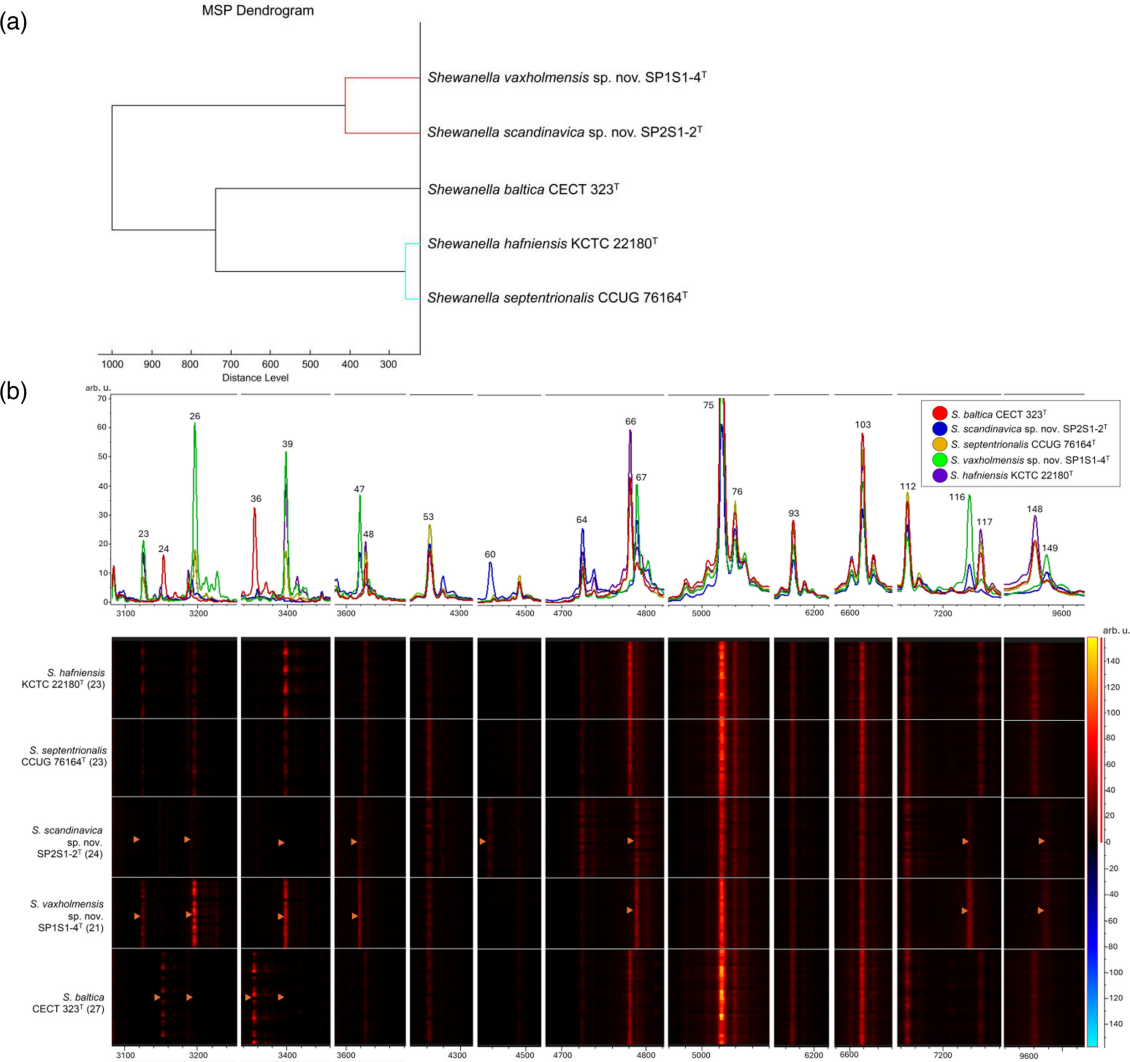
(**a**) Dendrogram of the MALDI-TOF mass spectral profiles obtained from the *Shewanella i*solates. The distance is displayed in relative units. (**b**) Unique peaks in spectral view and normalized gel peak view. The identified discriminating peaks between *Shewanella* strains are marked, with numerical peak numbers correlating to the peak statistics table (Table S1) and the orange arrows.

To dissect the proteomic differences in this group of strains, a protein profiling analysis of SP1S1-4^T^ and SP2S1-2^T^, in comparison to the type strains of *S. baltica*, *S. hafniensis*, and *S. septentrionalis*, was conducted using MALDI-TOF MS. First, to assess and curate interspecific spectral reproducibility, a CCI database was created and used to try to differentiate the type strain of *S. baltica* from the type strains of *S. hafniensis* and *S. septentrionalis*. However, matched CCI scores, as summarized in Table S4, showed that the *S. baltica* protein profile had certain interspecific similarity to those of both *S. hafniensis* and *S. septentrionalis*, except for a subset of identified unique peaks that gave it a lower CCI score. The type strain of *S. baltica* could be confidently discriminated from strains SP1S1-4^T^ and SP2S1-2^T^ based on the consistently low CCI match scores. It was not possible to differentiate *S. hafniensis* and *S. septentrionalis* based on the spectral profile data, but strains SP1S1-4^T^ and SP2S1-2^T^ were relatively distinguishable from all the other type strains and each other. Further, the dendrogram reconstructed using the MSP database, presented in Fig. 3a, showed distinct clustering of SP1S1-4^T^ and SP2S1-2^T^ in relation to the type strains of *S. baltica*, *S. hafniensis* and *S. septentrionalis*. MSP libraries were further analysed using multivariate cluster analysis. The resulting PCA with unit variance scaling, shown in Fig. S5, showcased the clear discrimination achieved for SP1S1-4^T^ and SP2S1-2^T^ with respect to the other type strains, as well as one from another. Representative spectra of the MSP library are presented in Fig. S6.

A heatmap of the MALDI-TOF normalized peak clustering is shown in Fig. 3b, with spectral view resolution of peaks to demonstrate their spectral intensity. Here**,** the distinctive peaks based on spectral intensity and mass-to-charge ratio (m/z) between the five strains are noted based on the top 20 significant peaks, identified by peak indexes in the spectral view and the presence or absence of peaks between species in the heatmap of normalized peaks. Additional peak statistics are available herewith as supplemental File S1. Despite their genomic distinctiveness, no distinguishing proteomic features were observed for the type strains of *S. hafniensis* and *S. septentrionalis*, except for the peaks that were present or absent in the type strain of *S. baltica*, as discussed. Unique differences at peaks 24 (3152.43 m/z) and 36 (3355.3 m/z) distinguished the type strain of *S. baltica* from the other *Shewanella* strains. The absence of signals at peaks 26 (3195.84 m/z) and 39 (3398.21 m/z), which had high overall intensity for the rest of the strains, further distinguished *S. baltica*. On the other hand, SP1S1-4^T^ and SP2S1-2^T^ showed significant differences at peaks 47 (3618.69 m/z), 67(4789.13 m/z) and 116(7237.57 m/z), and at a lower intensity, at peak 149 (9575.61 m/z) compared to the other *Shewanella* strains. Peaks 23, 26 and 39, at m/z 3124.89, 3195.81, and 3398.21, respectively, further differentiated between SP1S1-4^T^ and SP2S1-2^T^. In addition, although at a lower intensity, peak 60 (4458.07 m/z) was only present for SP2S1-2^T^. On the other hand, peak 105 (6617.82 m/z) was highly intense and shared by all tested *Shewanella* strains. Other peaks such as peaks 64, 93, 103, and 112, had generally significant DAve values, but they were also shared by all strains. Taken together, MALDI-TOF MS could discriminate the protein profiles of SP1S1-4^T^ and SP2S1-2^T^ from those of their closest genotypic relatives and one from another.

### Phenotypic features

We continued our study by characterizing the phenotypic properties of SP1S1-4^T^ and SP2S1-2^T^. Both strains were able to grow in Miller’s LB broth or LB agar, in marine broth or marine agar, and on blood agar medium. No haemolysis was detected on blood agar for either strain. The two strains formed convex, smooth colonies with the archetypal orange colour of *Shewanella* species when cultivated on LB agar (Fig. S7A-B). Cells grown on LB agar were scraped, suspended in PBS, and Gram-stained. Microscopic visualization of stained cells revealed Gram-negative bacilli (data not shown). Further examination of cellular morphological features was performed by SEM (Fig. S7C-D). SP1S1-4^T^ and SP2S1-2^T^ cells showed similar sizes, with SP1S1-4^T^ being 2.05 µm long and 0.57 µm wide, and SP2S1-2^T^ being 2.23 µm long and 0.64 µm wide, based on average measurements taken from 15 arbitrarily chosen single cells of each strain. At several instances, a single polar flagellum could be observed for both strains (Figs S7C and D), which is a typical feature of *Shewanella* [50]. Distinctively, cells of strain SP2S1-2^T^ presented a rough cellular morphotype (Fig. S7D).

The growth capacity of the six strains was tested on LB agar at different temperatures, as follows: 4, 23, 28, 30, 32, 35, 37, and 42 °C. Satisfactory growth was documented for all strains in the range 4–32 °C, with optimal growth at 23–30 °C. Conversely, growth was substantially impaired at 35 °C for SP2S1-2^T^ but not for SP1S1-4^T^. At 37 °C or higher, growth of both strains was poor or completely supressed. Proficient anaerobic growth, tested at 28 °C on Miller’s LB agar plates, was documented for both strains. The capacity of the isolates to grow at different NaCl concentrations was tested on LB agar supplemented with 0, 1.0, 2.5, 5.0, 7.5, or 10.0 % NaCl, at 28 °C. Growth in the range 0–2.5 % NaCl was satisfactory for both strains. Growth of both strains in the presence of 5.0 % NaCl was negligible, and no growth was recorded at 7.5 or 10.0 % NaCl. Growth at different pH values (pH 4.5–10.5, at 1.0 pH unit intervals) was tested in buffered LB broth at 28 %. The growth curves of the isolates are presented in Figs S7E–F and clearly evidence a similar capacity of both strains to grow in a pH range between pH 6.5 and 8.5. Outside of this pH range, growth was negligible or not detected (data not shown).

Given the close ecological and genomic relatedness of the strains under study, a comprehensive characterization of metabolic features was conducted following the CCUG NFX worksheet, and the data were compared to those previously determined for their closest genotypic relatives, namely, in order of phylogenetic relatedness, *S. septentrionalis* CCUG 76164^T^, *S. baltica* CCUG 39356^T^, and *S. hafniensis* KCTC 22180^T^ [13] (Table S5). A summary of the distinctive features that differentiate SP1S1-4^T^ and SP2S1-2^T^ from the type strains of other closely related *Shewanella* species is presented in Table 2. Distinctively, strain SP1S1-4^T^ was capable of maltose fermentation, a characteristic that was shared only with *S. baltica* CCUG 39356^T^. Aesculin hydrolysis was negative for both strains under investigation, in contrast to the reference type strains, that were positive.

**Table 2.** Phenotypic features that differentiate strains SP1S1-4^T^ and SP2S1-2^T^ from other closely related *Shewanella* species Strains: 1, *Shewanella vaxholmensis* sp. nov. SP1S1-4^T^ (=CCUG 76453^T^); 2, *Shewanella scandinavica* sp. nov. SP2S1-2^T^ (=CCUG 76457^T^); 3, *Shewanella septentrionalis* CCUG 76164^T^; 4, *Shewanella baltica* CCUG 39356^T^; 5, *Shewanella hafniensis* KCTC 22180^T^. ++, Rapidly positive; +, positive; −, negative; 0, test not done.

Test panel	Phenotypic test	1	2	3	4	5
OX	Catalase	−	−	−	−	+
OF	OF-maltose	+	−	−	++	−
DEC	Lysine, LD	−	−	−	0	−
	Ornithine, OD	++	++	++	0	−
ESC	ONPG	−	−	−	++	−
ASSIM	Sucrose	−	−	++	++	−
	Lactate	++	++	++	−	++
API 20NE	Aesculin	−	−	+	+	+
	d-Glucose	+	+	−	++	++
	Caprate	++	−	−	−	−
	Adipate	−	+	−	−	−
	Citrate	++	++	++	++	−
API ZYM	Esterase (C4)	−	+	++	+	−
	Ester lipase (C8)	−	+	++	+	−
	Trypsin	−	−	++	−	+
	Chymotrypsin	−	−	+	−	−
	Phosphoamidase	−	+	++	−	+
	*N*-Acetyl-β-glucosaminidase	++	−	++	++	++

Differences in the assimilation of certain carbon sources were recorded for SP1S1-4^T^ and SP2S1-2^T^ in comparison to the type strains of related species. As opposed to the type strain of *S. septentrionalis*, their closest genotypic relative, SP1S1-4^T^ and SP2S1-2^T^ tested negative for sucrose assimilation and positive for the assimilation of glucose. In the commercial API 20NE strip, assimilation of adipate was a unique characteristic of strain SP2S1-2^T^ in comparison to any other tested strain, whereas SP1S1-4^T^ showed a distinct capacity to assimilate caprate, in contrast to any other species. Both strains were negative for trypsin and chymotrypsin activities. In contrast, the type strain of *S. septentrionalis* tested positive for all the afore-mentioned reactions.

### Taxonomic conclusions

#### SP2S1-2^T^ and SP1S1-4^T^ represent two novel genospecies of *Shewanella* within a ‘species complex’

Taken together, our polyphasic approach, based on extensive genomic and phenotypic analyses, suggests the existence of two novel species within the genus *Shewanella* as per application of current taxonomic standards. The proposed names are *Shewanella vaxholmensis* sp. nov., the type strain being SP1S1-4^T^, and *Shewanella scandinavica* sp. nov., the type strain being SP2S1-2^T^. The proposed novel species exhibit high genomic, genotypic, proteomic, and phenotypic similarities to *S. septentrionalis*, *S. baltica* and *S. hafniensis*. Subtle phenotypic differences, which, nevertheless, could potentially be attributed to natural strain variability, distinguish the proposed novel species from *S. septentrionalis*. Genospecies that cannot be clearly differentiated from closely related species based on phenotypic traits are traditionally referred to as ‘genomovars’ [51]. However, as genomovars formally represent distinct genomic species, and for the sake of phylogenetic congruence (that is, given the close phylogenetic relatedness of the proposed novel species to both *S. septentrionalis* and *S. baltica*, see e.g. Fig. S1), we have avoided such a terminology.

Indeed, the two novel species described here coexist with *S. septentrionalis*, previously retrieved from a water sample collected in Vaxön [13], and with *S. baltica*, which is frequently isolated in our field samplings at this site and throughout the Baltic Sea. *S. hafniensis*, originally isolated from Baltic Sea fish [52], may share the same habitat as well. Ecological differentiation, DNA recombination, and horizontal acquisition of genes shape bacterial genomes, thus acting as major drivers of speciation in bacterial populations [53,54]. Outstanding levels of recombination have been reported for aquatic populations of *S. baltica* inhabiting the redox-stratified Baltic Sea water column [55]. Even higher levels of genetic exchange might exist in bacterial populations inhabiting soils or sediments, where bacteria might migrate across distinct microniches, encountering and mixing with other bacterial populations in the process [56]. There is evidence in favour [57] and against this hypothesis [58]. It remains an open question whether the genomic similarities between *S. septentrionalis*, *S. scandinavica* sp. nov., *S. vaxholmensis* sp. nov. and *S. baltica* could be a consequence of sympatric recombination in sediments of this geographical region, among other potential contributing factors. The phylogenomic evidence presented here suggests that these species form a ‘species complex’ of phenotypically and genetically related taxa, characterized by distinct lineages that form clusters separated by ANIb values ≥96 %. At this point, we intentionally quote the term ‘species complex’ to briefly reopen a controversial question in bacterial systematics.

#### Is there a continuum of diversity or are there genomic boundaries defining species?

Sampling biases are a major confounding factor in bacterial population genomics and taxonomic studies, with a tendency to favour the selection of genome sequences representing the most distinct lineages [56]. In the preparation of this work, we had sequenced the genomes of additional *Shewanella* isolates from the same sedimental habitat, named SP2S2-6, SP1S2-4, SP1S1-7, and SP2S2-4, each of which was initially identified as a potential representative of a novel genomic species of *Shewanella* based on their pairwise dDDH and ANIb values, which were <70 % and <96 %, respectively, with respect to the type strains of *S. septentrionalis*, *S. baltica*, and *S. hafniensis*, their closest genotypic relatives, as well as each one another (exemplified in Table S6 with pairwise ANIb values). A phylogenomic reconstruction incorporating these additional isolates, based on dDDH as implemented in TYGS, is presented in Fig. S8. From this phylogeny it is evident that, while SP2S2-6, SP1S2-4, SP1S1-7, and SP2S2-4 could represent novel taxa, there is limited phylogenomic confidence as inferred by node support values, and additional isolates would be needed to define potential genomic clusters and, therefore, genospecies [51]. For this reason, we have adopted a conservative position and refrained from presenting them as novel *Shewanella* species for the moment. The data presented here are compatible with a model in which a genomic discontinuity around ANIb values of 96 % define species clusters. However, this observation should not be taken as a categorical statement, as, certainly, a small representation of genomes is insufficient to draw such conclusions. Further sampling and sequencing efforts are necessary to gain a comprehensive understanding of strain and species diversity within this clade. This will elucidate the potential occurrence of a continuum of genomic diversity, which prompts us to question: what would, then, define a ‘species’? [59,60].

## Description of *Shewanella scandinavica* sp. nov.

*Shewanella scandinavica* (scan.di.na’vi.ca. N.L. fem. adj. *scandinavica*, inflected form of scandināvicus, ‘Scandinavia’, because of its geographical origin).

Cells are rod-shaped, facultative anaerobic, Gram-negative, and motile. On average, cells are 2.23 µm long and 0.64 µm wide. Growth is satisfactory in Miller’s LB agar, where it forms smooth, convex, circular colonies. Growth is also satisfactory on marine agar, blood agar, and Lyngby’s iron agar, where it forms black deposits because of its capacity to produce H_2_S. The type strain does not produce haemolysis on blood agar. Growth of the type strain occurs optimally at pH 6.5–8.5, and at 4–30 °C (optimally at 23–30 °C). The type strain does not require NaCl for growth and is slightly halotolerant (up to 4.5 % NaCl). The type strain is catalase-negative and positive for oxidase, ornithine decarboxylase, and DNAse reactions, can hydrolyse Tween 80 and gelatin, and can reduce nitrate and nitrite.

The type strain can assimilate lactate, and lactate+methionine, but not trehalose, arginine, norleucin or sucrose. Oxidation-*versus*-fermentation tests for the type strain are negative for maltose, d-glucose, adonitol, d-fructose and d-xylose.

In the commercial API 20 NE strip, the type strain is positive for assimilating glucose, *N*-acetyl glucosamine, maltose, gluconate, adipate, malate, and citrate. Negative reactions are recorded for tryptophanase, glucose fermentation, arginine dihydrolase, urease, aesculin hydrolysis, PNPG β-galactosidase, and the assimilation of the following carbon sources: l-arabinose, d-mannose, d-mannitol, caprate and phenylacetate.

In the commercial API ZYM strip, the type strain is positive for the following enzymatic activities: alkaline phosphatase, esterase (C4), ester lipase (C8), leucine arylamidase, and phosphoamidase. Negative activities are recorded for lipase (C14), valine arylamidase, cysteine arylamidase, trypsin, chymotrypsin, acid phosphatase, α-galactosidase, β-galactosidase, β-glucuronidase, α-glucosidase, β-glucosidase, *N*-acetyl-β-glucosaminidase, α-mannosidase and α-fucosidase.

The type strain is SP2S1-2^T^ (CCUG 76457^T^=CECT 30688^T^), isolated from sediments collected in Vaxholm, Sweden. The size of its draft genome sequence is 5 041 805 bp, with a G+C content of 46.3 mol% (GenBank accession: JAUOES000000000). Its nearly complete 16S rRNA sequence (1401 bp) is available from GenBank under accession OP758209. Other whole genome sequenced strains belonging to this species are VAX-SP4-0CM-4 (GenBank accession: JBCHKS000000000) and VAX-SP4-0CM-7 (GenBank accession: JBCHKR000000000).

## Description of *Shewanella vaxholmensis* sp. nov.

*Shewanella vaxholmensis* (vax.holm.en’sis. N.L. fem. adj. *vaxholmensis*, from Vaxholm, a town located on the island of Vaxön, in the Stockholm archipelago).

Cells are rod-shaped, facultative anaerobic, Gram-negative, and motile. On average, cells are 2.05 µm long and 0.57 µm wide. Growth is satisfactory in Miller’s LB agar, where it forms smooth, convex, circular colonies. Growth is also satisfactory in marine agar, blood agar, and Lyngby’s iron agar, where it forms black deposits because of its capacity to produce H_2_S. The type strain does not produce haemolysis on blood agar. Growth of the type strain occurs optimally at pH 6.5–8.5, and at 4–35 °C (optimally at 23–30 °C). The type strain does not require NaCl for growth and is slightly halotolerant (up to 2.5 % NaCl). The type strain is catalase-negative and positive for oxidase, ornithine decarboxylase, and DNAse reactions, can hydrolyse Tween 80 and gelatin, and can reduce nitrate and nitrite.

The type strain is positive for assimilating lactate and lactate+methionine, but not for trehalose, arginine, norleucin or sucrose. Oxidation-*versus*-fermentation tests for the type strain are positive for maltose and negative for d-glucose, adonitol, d-fructose, and d-xylose.

In the commercial API 20 NE strip, the type strain is positive for assimilating glucose, *N*-acetyl glucosamine, maltose, gluconate, caprate, malate, and citrate, as well as gelatinase activity. For the type strain, negative reactions are recorded for tryptophanase, glucose fermentation, arginine dihydrolase, urease, aesculin hydrolysis, PNPG β-galactosidase, as well as the assimilation of the following carbon sources: l-arabinose, d-mannose, d-mannitol, adipate, and phenylacetate.

In the commercial API ZYM strip, the type strain is positive for the following enzymatic activities: alkaline phosphatase, leucine arylamidase, and *N*-acetyl-β-glucosaminidase. Negative for esterase (C4), ester lipase (C8), lipase (C14), cysteine arylamidase, trypsin, chymotrypsin, acid phosphatase, phosphoamidase, α-galactosidase, β-galactosidase, β-glucuronidase, α-glucosidase, β-glucosidase, α-mannosidase and α-fucosidase activities.

The type strain is SP1S1-4^T^ (CCUG 76453^T^=CECT 30684^T^), isolated from sediments collected in Vaxholm, Sweden. The size of its draft genome sequence is 4 920 147 bp, with a G+C content of 46.0 mol% (GenBank accession: JAUOEV000000000). Its nearly complete 16S rRNA sequence (1481 bp) is available from GenBank under accession OP758205. Other whole genome sequenced strains belonging to this species are VAX-SP0-0CM-1 (GenBank accession: JBCHKU000000000) and VAX-SP0-4CM-4 (GenBank accession: JBCHKT000000000).

## supplementary material

10.1099/ijsem.0.006480 Uncited Supplementary Material 1.

10.1099/ijsem.0.006480 Uncited Supplementary Data Sheet 1.

## Abbreviations

ANIb: blast-based average nucleotide identity
ANIm: ANI based on the MUMmer algorithm
BTS: bacterial test standard
CCI: composite correlation index
dDDH: digital DNA–DNA hybridization
GBDP: genome blast distance phylogeny
LB: lysogeny broth
MALDI-TOF MS: matrix-assisted linear desorption/ionization-time-of-flight mass spectrometry
MP: maximum-parsimony
MSP: main spectra profile
NJ: neighbour-joining
OGRI: overall genome-relatedness index
PCA: principal component analysis
SEM: Scanning Electron Microscopy
TYGS: Type (Strain) Genome Server
WGS: whole-genome sequence
