# Supplementary material for: A hotspot of diversity: novel Shewanella species isolated from Baltic Sea sediments delineate a sympatric species complex
Source: Int J Syst Evol Microbiol. 2024 Aug 16;74(8):006480. doi: 10.1099/ijsem.0.006480 (PMC11329295; doi:10.1099/ijsem.0.006480)

A hotspot of diversity: novel *Shewanella* species isolated from Baltic Sea sediments delineate a sympatric species complex

Alberto J. Martín-Rodríguez[1,2*], Víctor Fernández-Juárez[3,4], Valerie D. Valeriano[2], Indiwari Mihindukulasooriya[2], Livia Ceresnova[2], Enrique Joffré[2,5], Susanne Jensie-Markopoulos[4,6], Edward R. B. Moore[3,4,6], Åsa Sjöling[2,5]

[1] Department of Clinical Sciences, University of Las Palmas de Gran Canaria, Las Palmas de Gran Canaria, Spain

[2] Centre for Translational Microbiome Research (CTMR), Department of Microbiology, Tumor and Cell Biology, Karolinska Institute, Stockholm, Sweden

[3] Department of Infectious Diseases, Institute for Biomedicine, Sahlgrenska Academy of the University of Gothenburg, Gothenburg, Sweden

[4] Culture Collection University of Gothenburg (CCUG), Sahlgrenska University Hospital and Sahlgrenska Academy of the University of Gothenburg, Gothenburg, Sweden

[5] Department of Chemistry and Molecular Biology (CMB) University of Gothenburg, Gothenburg, Sweden

[6] Department of Clinical Microbiology, Sahlgrenska University Hospital, Region Västra Götaland, Gothenburg, Sweden

Correspondence:

Alberto J. Martín-Rodríguez alberto.martin@ulpgc.es; jonatan.martin.rodriguez@ki.se

-SUPPLEMENTARY MATERIAL-

Table S1. Pairwise dDDH comparisons between SP1S1-4^T, SP2S1-2^T, and reference *Shewanella* type strain genomes. C.I. = confidence interval; d_4 refers to the dDDH calculation formula, as implemented in TYGS.

Query strain	Subject strain	dDDH (d_4, %)	C.I. (d_4, %)	G+C content difference (%)
SP1S1-4^T	SP2S1-2^T	64.9	[62.0 - 67.8]	0.24
SP1S1-4^T	*Shewanella septentrionalis* SP1W3^T	64.8	[61.8 - 67.6]	0.06
SP2S1-2^T	*Shewanella septentrionalis* SP1W3^T	63.8	[60.9 - 66.7]	0.3
SP2S1-2^T	*Shewanella baltica* NCTC 10735^T	63.2	[60.3 - 66.0]	0.06
SP1S1-4^T	*Shewanella baltica* NCTC 10735^T	61.3	[58.5 - 64.1]	0.3
SP1S1-4^T	*Shewanella hafniensis* ATCC BAA-1207^T	60.3	[57.5 - 63.1]	0.22
SP2S1-2^T	*Shewanella hafniensis* ATCC BAA-1207^T	59.1	[56.3 - 61.9]	0.02
SP1S1-4^T	*Shewanella oncorhynchi* S-1^T	40.4	[37.9 - 42.9]	0.79
SP2S1-2^T	*Shewanella oncorhynchi* S-1^T	40.3	[37.8 - 42.8]	1.03
SP1S1-4^T	*Shewanella glacialipiscicola* T147^T	28.9	[26.5 - 31.4]	1.73
SP2S1-2^T	*Shewanella glacialipiscicola* T147^T	28.7	[26.4 - 31.2]	1.97
SP2S1-2^T	*Shewanella morhuae* ATCC BAA-1205^T	28.1	[25.7 - 30.6]	2.31
SP1S1-4^T	*Shewanella morhuae* ATCC BAA-1205^T	27.9	[25.5 - 30.3]	2.07
SP1S1-4^T	*Shewanella putrefaciens* NBRC 3908^T	26.6	[24.2 - 29.1]	1.72
SP1S1-4^T	*Shewanella putrefaciens* JCM 20190^T	26.6	[24.2 - 29.1]	1.74
SP2S1-2^T	*Shewanella putrefaciens* JCM 20190^T	26.5	[24.1 - 29.0]	1.98
SP1S1-4^T	*Shewanella profunda* DSM15900^T	26.5	[24.2 - 29.0]	1.11
SP2S1-2^T	*Shewanella putrefaciens* NBRC 3908^T	26.5	[24.2 - 29.0]	1.96
SP2S1-2^T	*Shewanella profunda* DSM15900^T	26.4	[24.1 - 28.9]	1.35
SP2S1-2^T	*Alteromonas sediminis* U0105^T	23.1	[20.8 - 25.6]	1.01
SP1S1-4^T	*Alteromonas sediminis* U0105^T	22.7	[20.4 - 25.2]	0.77
SP2S1-2^T	*'Shewanella cutis'* PS-2^T	22.6	[20.3 - 25.0]	0.28
SP1S1-4^T	*'Shewanella cutis'* PS-2^T	22.5	[20.2 - 25.0]	0.04
SP2S1-2^T	*Shewanella xiamenensis* JCM16212^T	22.5	[20.2 - 25.0]	0.03
SP2S1-2^T	*Shewanella decolorationis* S12^T	22.4	[20.1 - 24.8]	0.81
SP1S1-4^T	*Shewanella xiamenensis* JCM16212^T	22.4	[20.1 - 24.8]	0.21
SP1S1-4^T	*Shewanella mangrovisoli* FJAT-51754^T	22.4	[20.1 - 24.8]	2.16
SP2S1-2^T	*Shewanella mangrovisoli* FJAT-51754^T	22.3	[20.1 - 24.8]	1.92
SP1S1-4^T	*Shewanella decolorationis* S12^T	22.3	[20.0 - 24.8]	1.05
SP1S1-4^T	*'Shewanella shenzhenensis'* A25^T	21.5	[19.3 - 23.9]	1.37
SP2S1-2^T	*'Shewanella shenzhenensis'* A25^T	21.5	[19.2 - 23.9]	1.13
SP1S1-4^T	*'Shewanella salipaludis'* SHSM-M6^T	19.6	[17.4 - 22.0]	9.66
SP2S1-2^T	*'Shewanella salipaludis*' SHSM-M6^T	19.4	[17.2 - 21.8]	9.42

Table S2. ANIb matrix. Reciprocal pairwise comparisons between novel and reference *Shewanella* type strain genome sequences are shown. The percentage of aligned nucleotides in each comparison is indicated in parentheses.

	Shewanella baltica **NCTC10735^T**	***Shewanella hafniensis*** **ATCC BAA-1207^T**	***Shewanella septentrionalis*** **SP1W3^T**	**SP1S1-4^T**	**SP2S1-2^T**
Shewanella baltica **NCTC10735^T**	*	93.47 *(78.51)*	94.76 *(79.01)*	94.93 *(78.77)*	95.28 *(80.74)*
Shewanella hafniensis **ATCC BAA-1207^T**	93.55 *(83.08)*	*	94.92 *(82.64)*	94.73 *(82.21)*	94.54 *(83.75)*
Shewanella septentrionalis **SP1W3^T**	94.75 *(80.04)*	94.88 *(78.37)*	*	95.51 *(83.92)*	95.39 *(81.66)*
**SP1S1-4^T**	94.93 *(83.92)*	94.69 *(82.50)*	95.63 *(87.60)*	*	95.53 *(86.16)*
**SP2S1-2^T**	95.25 *(84.77)*	94.47 *(82.21)*	95.37 *(84.53)*	95.49 *(84.04)*	*

Table S3. AAI matrix. Pairwise comparisons between novel and reference type strains are indicated.

	***Shewanella baltica* NCTC10735^T**	***Shewanella hafniensis* ATCC BAA-1207^T**	***Shewanella septentrionalis* SP1W3^T**	**SP1S1-4^T**	**SP2S1-2^T**
***Shewanella baltica* NCTC10735^T**	*				
***Shewanella hafniensis* ATCC BAA-1207^T**	96.55	*			
***Shewanella septentrionalis* SP1W3^T**	97.16	97.28	*		
**SP1S1-4^T**	97.45	97.20	97.54	*	
**SP2S1-2^T**	97.45	97.08	97.54	97.53	*

Table S4. Composite Correlation Index (CCI) delineating similarity indices between spectral peaks. Green: high similarity confidence; Yellow: acceptable similarity confidence; Red: low similarity confidence. Numerical values depict highest CCI scores achieved in each matching.

	Shewanella baltica **CECT 323^T**	***Shewanella hafniensis*** **KCTC 22180^T**	***Shewanella septentrionalis*** **CCUG 76164^T**	**SP1S1-4^T**	**SP2S1-2^T**
Shewanella baltica **CECT 323^T (n=27)**	1.019				
Shewanella hafniensis **KCTC 22180^T (n=23)**	0.847	1.019			
Shewanella septentrionalis **CCUG 76164^T (n=23)**	0.847	1.001	1.019		
**SP1S1-4^T (n=21)**	0.721	0.849	0.865	1.019	
**SP2S1-2^T (n=24)**	0.662	0.837	0.868	0.876	1.019

Table S5. Phenotypic features of type strains of *Shewanella* spp.

Species: **1** *Shewanella vaxholmensis* sp. nov. SP1S1-4^T = CCUG 76453^T; **2**, *Shewanella scandinavica* sp. nov. SP2S1-2^T = CCUG 76457^T; **3**, *Shewanella septentrionalis* CCUG 76164^T; **4**, *Shewanella baltica* CCUG 39356^T **5**, *Shewanella hafniensis* KCTC 22180^T. ++, Rapidly positive; +, positive; −, negative; 0, test not done.

Test Panel	Phenotypic Test	1	2	3	4	5
**Ox**	Oxidase, TMPD	++	++	++	++	++
	Catalase	-	-	-	-	+
	Hemolysis, horse blood	-	-	-	-	-
	Penicillin, mm	-	-	-	-	-
	Blood 30°C - growth	++	++	++	++	++
	Blood 37°C - growth	-	-	-	-	-
	Drigalski - growth	++	++	-	+	++
	NA 30°C - growth	0	0	0	0	0
	NA 37°C - growth	0	0	0	0	0
	NA 42°C - growth	-	-	-	-	-
	Motility	++	++	0	0	0
**OF**	OF-D-Glucose	-	-	-	-	-
	OF-Maltose	+	-	-	++	-
	OF-Adonitol	-	-	-	-	-
	OF-D-Fructose	-	-	-	-	-
	OF-D-Xylose	-	-	-	-	-
	Fluorescin	-	-	-	-	-
	Centrimide	-	-	-	-	-
	10% Lactose	-	-	-	-	-
	Tween 80	++	++	++	++	++
	Amylase, MH	-	-	-	-	-
**DEC**	Lysine, LD	-	-	-	0	-
	Ornithine, OD	++	++	++	0	-
	Arginine, ADH	-	-	-	0	-
	NO_3^- reduction	++	++	++	++	++
	NO_2^- reduction	+	+	+	0	+
	Urease, Christ.	-	-	-	0	-
	Gelatine Kohn	++	++	++	0	++
**ESC**	ONPG	-	-	-	++	-
	Indole	-	-	-	-	-
	DNAse	+	++	++	+	++
	Acetamide	-	-	-	-	-
	Acid in TSI	-	-	-	-	-
	H_2S in TSI	++	++	++	++	++
	NaCl 0.5% - growth	++	++	++	++	++
	NaCl 1.5% - growth	++	++	++	++	++
	NaCl 3.0% - growth	-	+	++	++	+
	NaCl 4.5% - growth	-	+	-	-	-
	NaCl 6.0% - growth	-	-	-	-	-

**ASSIM**	Trehalose	-	-	-	-	-
	Arginine	-	-	-	-	-
	Norleucin	-	-	-	-	-
	Sucrose	-	-	++	++	-
	Lactate	++	++	++	-	++
	Lactate + Methionine	++	++	++	++	++
**API 20NE**	Tryptophanase	-	-	-	-	-
	Glucose fermentation	-	-	-	-	-
	Arg. dihydrol	-	-	-	-	-
	Urease	-	-	-	-	-
	Esculin	-	-	+	+	+
	Gelatinase	++	++	++	++	++
	PNPG β-gal	-	-	-	-	-
	D-Glucose	+	+	-	++	++
	L-Arabinose	-	-	-	-	-
	D-Mannose	-	-	-	-	-
	D-Mannitol	-	-	-	-	-
	N-Ac-glucosamine	++	++	++	++	++
	Maltose	++	++	++	++	++
	D-Gluconate	++	++	++	++	++
	Caprate	++	-	-	-	-
	Adipate	-	+	-	-	-
	L-Malate	++	++	++	++	++
	Citrate	++	++	++	++	-
	Phenylacetate	-	-	-	-	-
**ZYM**	Alkaline Phosphatase	++	++	++	++	++
	Esterase (C-4)	-	+	++	+	-
	Ester Lipase (C-8)	-	+	++	+	-
	Lipase (C-14)	-	-	-	-	-
	Leucine arylamidase	+	+	++	+	++
	Valine arylamidase	-	-	-	-	-
	Cysteine arylamidase	-	-	-	-	-
	Trypsin	-	-	++	-	+
	Chymotrypsin	-	-	+	-	-
	Acid Phosphatase	-	-	-	-	-
	Phosphoamidase	-	+	++	-	+
	α-galactosidase	-	-	-	-	-
	β-galactosidase	-	-	-	-	-
	β-glucuronidase	-	-	-	-	-
	α-glucosidase	-	-	-	-	-
	β-glucosidase	-	-	-	-	-
	N-Acetyl-β-gluc	++	-	++	++	++
	α-mannosidase	-	-	-	-	-
	α-fucosidase	-	-	-	-	-

Table S6. ANIb matrix of diverse *Shewanella* spp. retrieved from Baltic Sea sediments. Reciprocal pairwise comparisons between novel and reference *Shewanella* type strain genome sequences are shown. The percentage of aligned nucleotides in each comparison is indicated in parentheses.

	S. hafniensis ATCC BAA-1207^{T}	*S. septentrionalis* SP1W3^{T}	*S. baltica* NCTC10735^{T}	SP1S1-4^{T}	SP2S1-2^{T}	SP2S2-4	SP2S2-6	SP1S2-4	SP1S1-7
S. hafniensis ATCC BAA-1207^{T}	*								
S. septentrionalis SP1W3^{T}	94.88 [78.37]	*							
S. baltica NCTC10735^{T}	93.47 [78.51]	94.76 [79.01]	*						
SP1S1-4^{T}	94.69 [82.50]	95.63 [87.60]	94.93 [83.92]	*					
SP2S1-2^{T}	94.47 [82.21]	95.37 [84.53]	95.25 [84.77]	95.49 [84.04]	*				
SP2S2-4	94.61 [83.36]	95.40 [84.47]	95.24 [85.61]	95.56 [84.16]	95.86 [89.06]	*			
SP2S2-6	94.55 [82.88]	95.34 [84.62]	95.16 [85.29]	95.77 [85.17]	95.90 [89.58]	95.77 [88.40]	*		
SP1S2-4	94.63 [81.15]	95.26 [82.65]	95.09 [83.15]	95.63 [83.35]	95.84 [87.27]	95.75 [85.84]	95.84 [87.49]	*	
SP1S1-7	94.67 [82.06]	95.39 [82.71]	95.27 [84.21]	95.39 [82.60]	95.86 [87.60]	95.89 [87.84]	95.83 [86.92]	95.86 [88.57]	*

Figure S1. Genome sequence-based phylogeny showing the taxonomic relationships of strains SP1S1-4^T, SP2S1-2^T, VAX-SP0-0CM-1, VAX-SP0-4CM-4, VAX-SP4-0CM-4 and VAX-SP4-0CM-7 with respect to other *Shewanella* type strains. The tree was inferred with FastME 2.1.6.1 [19] from GBDP distances calculated from genome sequences. The branch lengths are scaled in terms of GBDP distance formula d_5 [8]. The numbers above branches are GBDP pseudo-bootstrap support values from 100 replications. Node support values ≥ 50 are indicated. The tree was rooted at the midpoint [20].

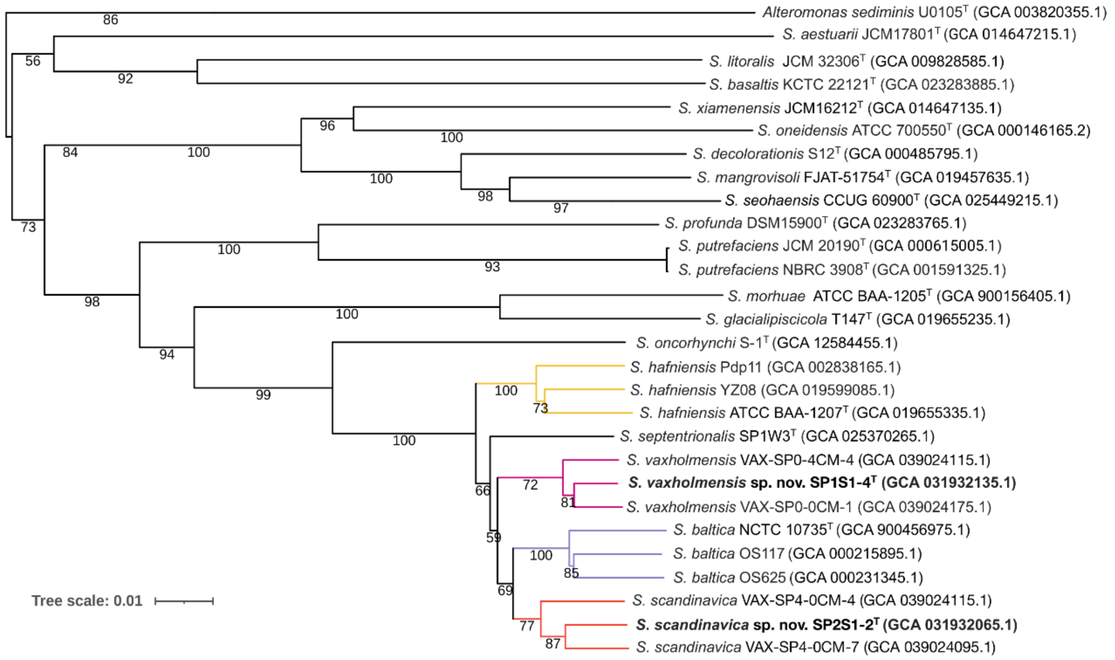

Figure S2. Maximum-likelihood phylogenetic reconstruction (HKY+G+I) based on partial 16S rRNA sequences. Numbers on the nodes indicate the percentages of 1,000 bootstrap replicates. Accessions of type strain 16S rRNA gene sequences are indicated in parentheses. The tree is drawn to scale, with branch lengths measured in the number of substitutions per site. The newly proposed species are highlighted in bold. Node support values ≥ 50 are indicated.

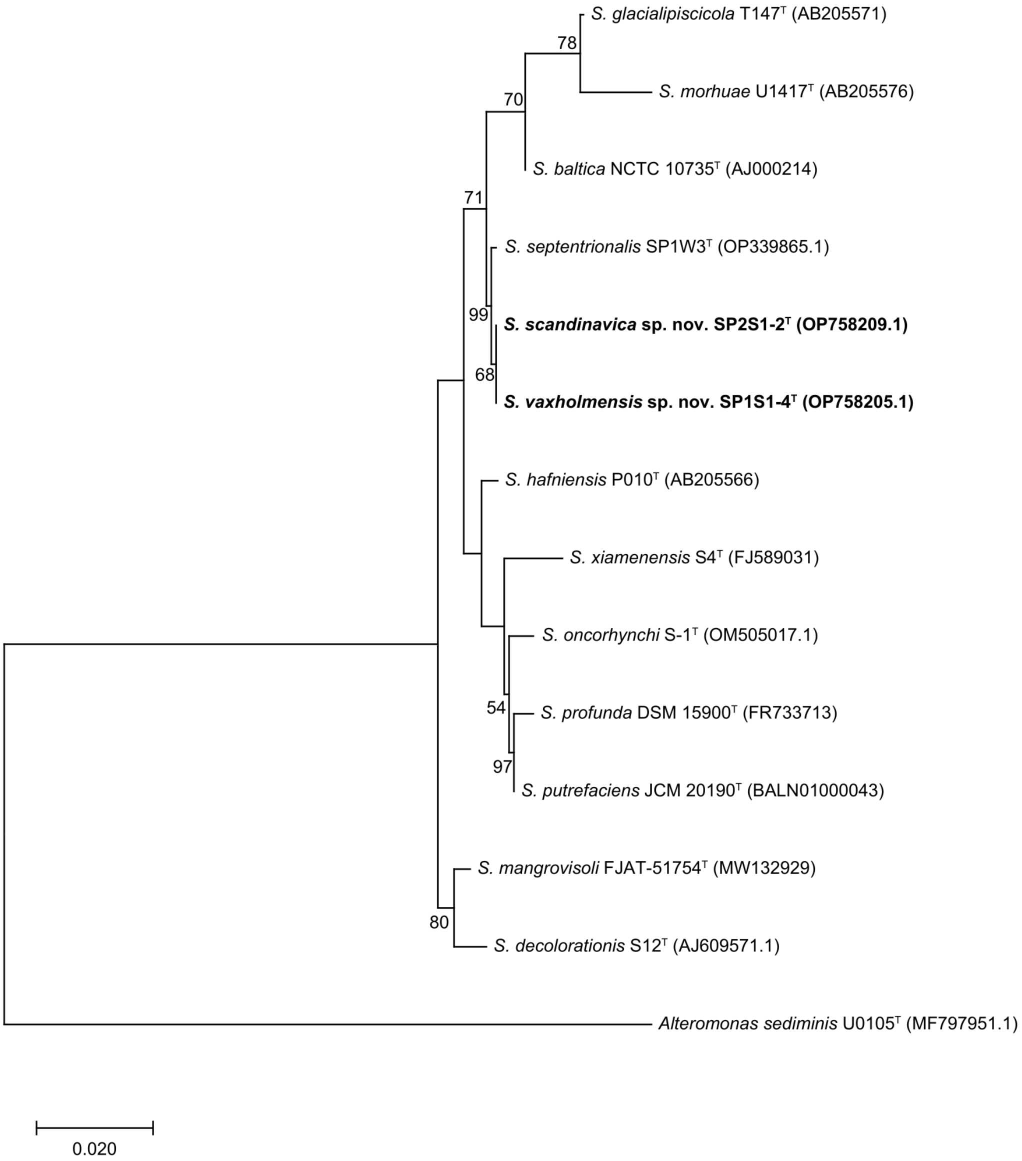

Figure S3. Neighbour-Joining phylogenetic reconstruction based on partial 16S rRNA sequences. Numbers on the nodes indicate the percentages of 1,000 bootstrap replicates. Accessions of type strain 16S rRNA gene sequences are indicated in parentheses. The tree is drawn to scale, with branch lengths measured in the number of substitutions per site. The newly proposed species are highlighted in bold. Node support values ≥ 50 are indicated.

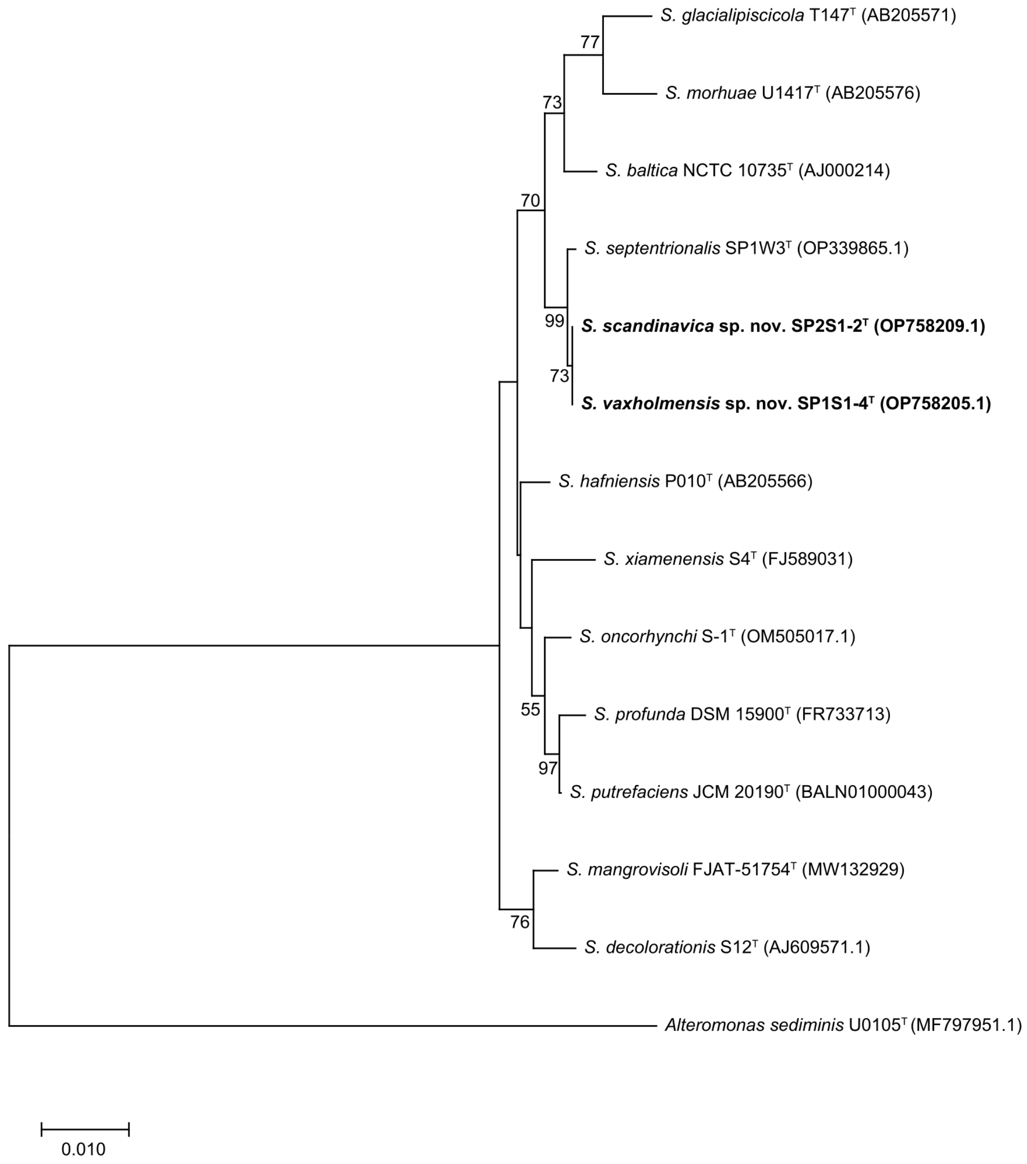

Figure S4. Evolutionary history inferred using the Maximum Parsimony method based on partial 16S rRNA sequences. Numbers on the nodes indicate the percentages of 1,000 bootstrap replicates. The MP tree was obtained using the Subtree-Pruning-Regrafting (SPR) algorithm, as implemented in MEGA X. Accessions of type strain 16S rRNA gene sequences are indicated in parentheses. The newly proposed species are highlighted in bold. Node support values ≥ 50 are indicated.

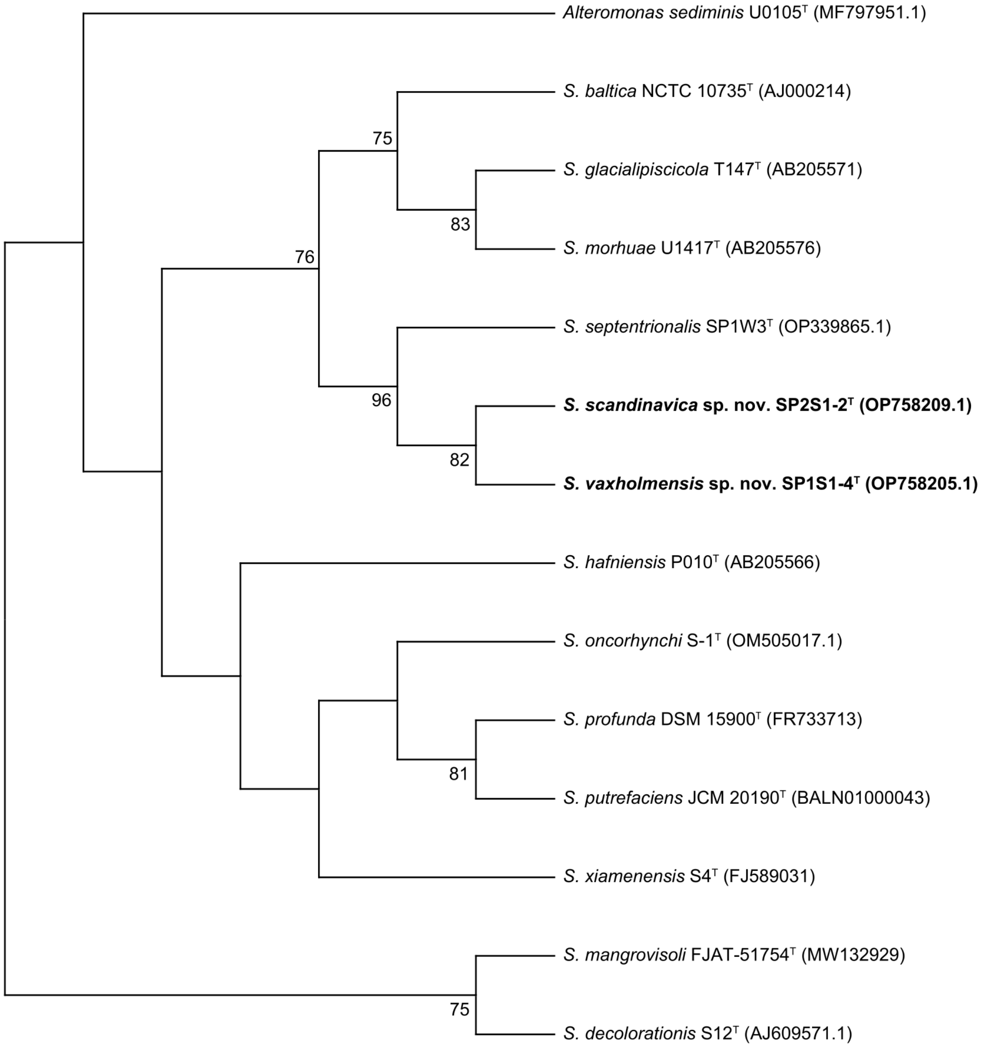

Figure S5. Principal component analysis (PCA) showing the distribution of the *Shewanella* strains (*S. baltica* CECT 323^{T}, *S. hafniensis* KCTC 22180, *S. septentrionalis* CCUG 76164, S. vaxholmensis sp. nov. SP1S1-4^{T} and *S. scandinavica* sp. nov. SP2S1-2^{T}) among the first three principal components (PCs) based on discriminating peaks presented in Figure 3B. PC1, PC2, and PC3 combined explain 70% total variance.

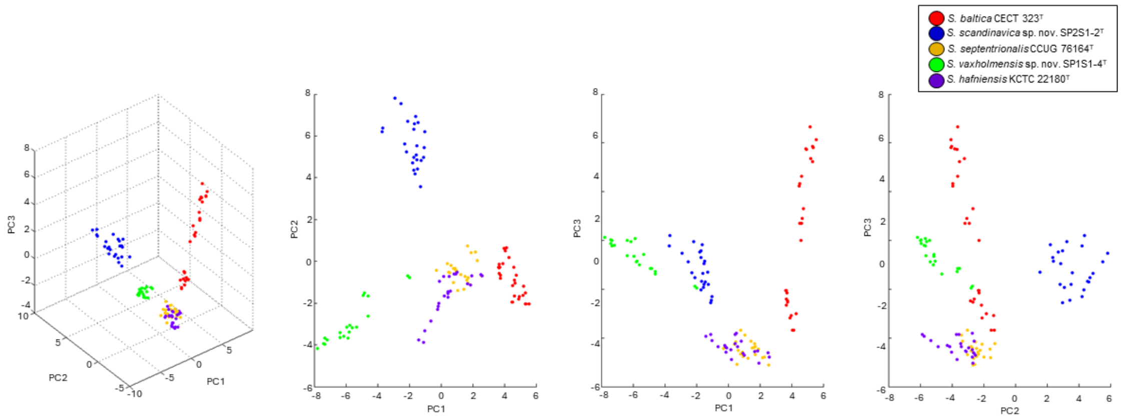

Figure S6. Automatic peak selection for Maldi-TOF identification with representative spectra. Selected peaks using the Bruker flexAnalysis software are presented in (m/z). The same peaks are used for identification of validation spectra using the MBT Compass Explorer Software.

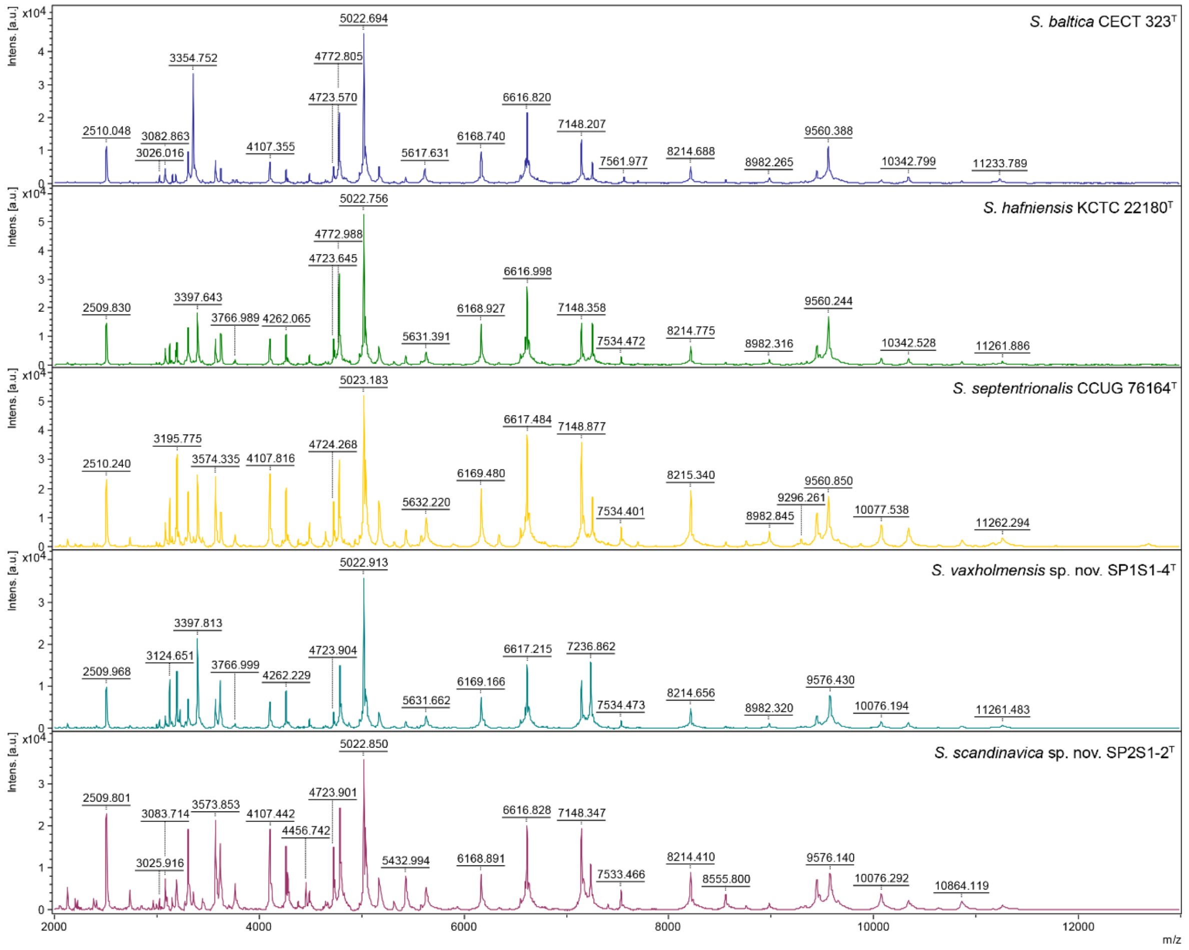

Figure S7. Phenotypic features of strains SP1S1-4^T and SP2S1-2^T. A-B. Macroscopic appearance of colonies grown on Miller's LB agar at 28 °C. **C-D.** High magnification SEM images of cells. Note the presence of single, polar flagella (arrows) and the rough surface of SP2S1-2^T cells in contrast to the smooth surface of SP1S1-4^T cells. **E-F.** Growth curves in buffered Miller's LB broth at 28 °C and variable pH. Shown are the curves for the pH values in which detectable growth was recorded.

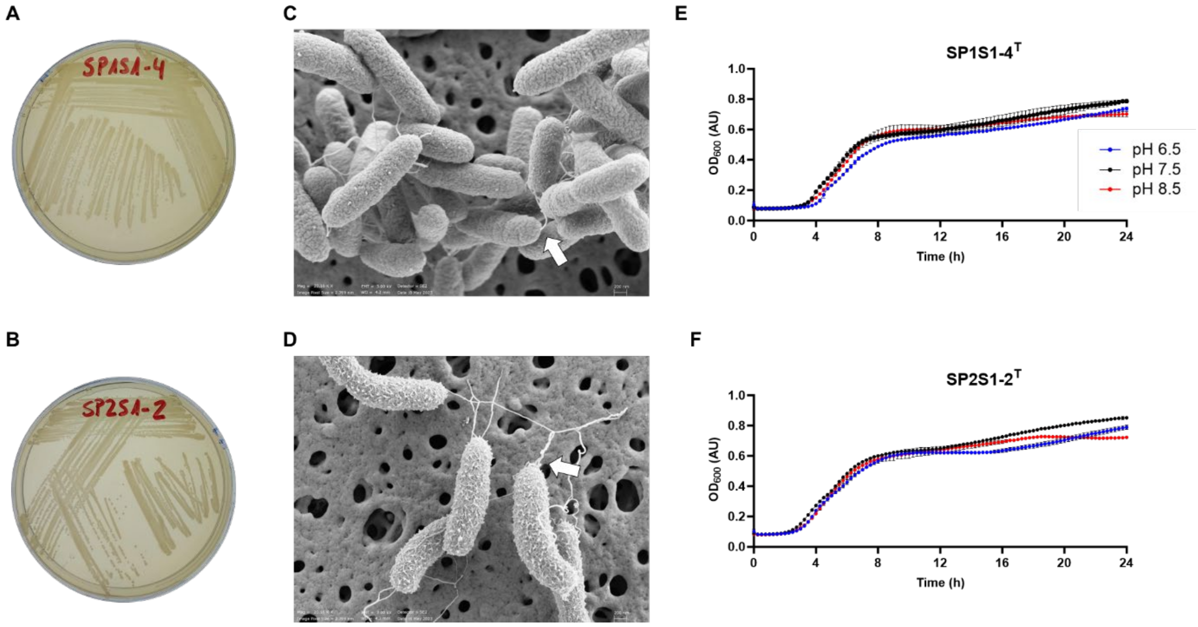

Figure S8. Phylogenomic GBDP tree inferred with FastME 2.1.6.1 from whole genome sequences of *Shewanella* spp. strains. The branch lengths are scaled in terms of GBDP distance formula d_5, as implemented in TYGS. The numbers above branches are GBDP pseudo-bootstrap support values from 100 replications. Node support values ≥ 50 are indicated. The tree was rooted at the midpoint. Clusters of proposed novel species are highlighted.

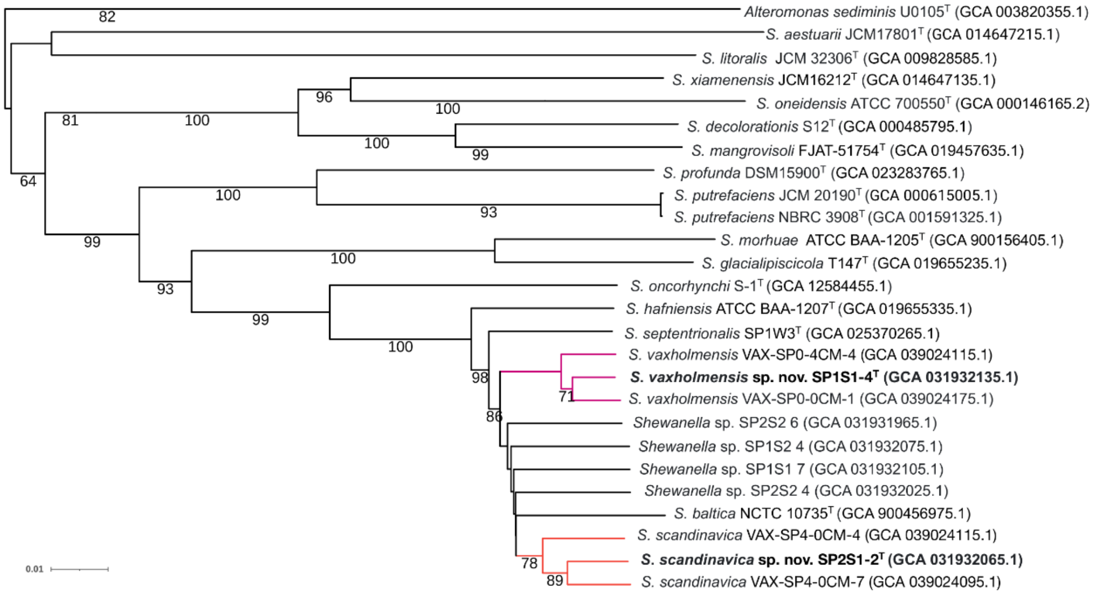

Supplement: Uncited Supplementary Material 1. [file ijsem-74-06480-s001.pdf]
